# dConsensus: a tool for displaying domain assignments by multiple structure-based algorithms and for construction of a consensus assignment

**DOI:** 10.1186/1471-2105-11-310

**Published:** 2010-06-09

**Authors:** Kieran Alden, Stella Veretnik, Philip E Bourne

**Affiliations:** 1 York Centre for Complex Systems Analysis (YCCSA), University of York, Heslington, York, YO10 5DD, UK; 2 San Diego Supercomputer Center, University of California San Diego, 9500 Gilman Dr., La Jolla, CA 92093-0743, USA; 3 Skaggs School of Pharmacy and Pharmaceutical Sciences, University of California San Diego, 9500 Gilman Dr., La Jolla, CA 92093-0636, USA

## Abstract

**Background:**

Partitioning of a protein into structural components, known as domains, is an important initial step in protein classification and for functional and evolutionary studies. While the systematic assignments of domains by human experts exist (CATH and SCOP), the introduction of high throughput technologies for structure determination threatens to overwhelm expert approaches. A variety of algorithmic methods have been developed to expedite this process, allowing almost instant structural decomposition into domains. The performance of algorithmic methods can approach 85% agreement on the number of domains with the consensus reached by experts. However, each algorithm takes a somewhat different conceptual approach, each with unique strengths and weaknesses. Currently there is no simple way to automatically compare assignments from different structure-based domain assignment methods, thereby providing a comprehensive understanding of possible structure partitioning as well as providing some insight into the tendencies of particular algorithms. Most importantly, a consensus assignment drawn from multiple assignment methods can provide a singular and presumably more accurate view.

**Results:**

We introduce dConsensus http://pdomains.sdsc.edu/dConsensus; a web resource that displays the results of calculations from multiple algorithmic methods and generates a domain assignment consensus with an associated reliability score. Domain assignments from seven structure-based algorithms - PDP, PUU, DomainParser2, NCBI method, DHcL, DDomains and Dodis are available for analysis and comparison alongside assignments made by expert methods. The assignments are available for all protein chains in the Protein Data Bank (PDB). A consensus domain assignment is built by either allowing each algorithm to contribute equally (simple approach) or by weighting the contribution of each method by its prior performance and observed tendencies. An analysis of secondary structure around domain and fragment boundaries is also available for display and further analysis.

**Conclusion:**

dConsensus provides a comprehensive assignment of protein domains. For the first time, seven algorithmic methods are brought together with no need to access each method separately via a webserver or local copy of the software. This aggregation permits a consensus domain assignment to be computed. Comparison viewing of the consensus and choice methods provides the user with insights into the fundamental units of protein structure so important to the study of evolutionary and functional relationships.

## Background

The process of partitioning a protein into structural components, known as domains, has been much studied since the partitioning concept was suggested over thirty years ago by Wetlaufer [1] and Rossman and Liljas [2]. The result of such partitioning impacts the classification of protein structures, and facilitates their further study [3]. Human expert methods, such as CATH [4], SCOP [5], and annotations provided by the structural biologist determining the structure, are used to define such structural units within a protein chain. However, with the introduction of high throughput technologies, protein structures are solved at a rate that vastly overwhelms human expert approaches [3]. As a result assignments of domains to newly solved protein structures lags at least 18 months behind, currently equating to approximately 10,000 structures.

Algorithms for partitioning tertiary structure into domains have been appearing steadily over the last thirty years or more [6]. Use of a reliable algorithm would mean protein domain annotation could be provided almost instantly. However, how to define a domain is a much debated topic; and as such the human expert's or algorithmic interpretation of this definition may have an effect on the domains produced by the assignment, and consequently effect classification of the protein structure universe [7]. Structural domains are typically defined using a set of characteristics such as: compactness, structural stability, presence of the hydrophobic core, folding independently of the rest of the protein, occurrence in combinations with a variety of other domains, and presence of function. However, there are many exceptions to these six criteria, thus even the expert methods cannot agree in difficult cases on partitioning of the structure. If such complex cases are excluded, an expert consensus benchmark dataset can be defined which is useful in assessing the performance of algorithmic domain assignment methods [7,8]. It was shown that the best performance of algorithmic methods is around 85% when complex cases - the ones that cause experts to disagree - are not included (unpublished results and [8]). Furthermore, each method has a somewhat different approach to the partitioning of the structure, thus different methods 'err' (disagree with expert consensus) on different structures [8].

To partially circumvent the issue of disagreement among algorithmic methods, it is suggested that the predictions from multiple algorithms be presented and compared, rather than relying on the prediction of a single algorithm. Further to this comparison, generation of a consensus assignment among a variety of methods has been suggested [6] to capture the most likely domain partitioning predicted by the majority of methods. The idea of reaching consensus among automatic domain assignment methods is not completely new. It was applied by Jones et al. [9] to three methods available at the time: DOMAK, PUU and DETECTIVE. The consensus approach became part of the domain assignment by experts in CATH [10].

**Figure 1:**
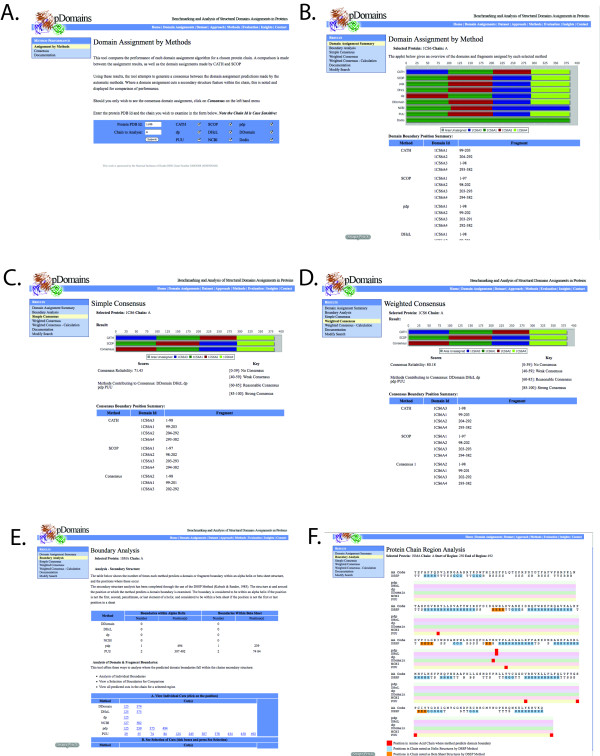
**Features of the dConsensus tool**. The screenshots represent a subset of the pages of dConsensus. **A**. Initial query form **B**. Results of domain assignments by all methods for 1cs6A. **C**. Consensus (simple) for 1cs6A **D**. Consensus (weighted) for 1cs6A. **E**. Boundary analysis options for 1smaA **F**. Boundary analysis of specified region of 1smaA. Alpha-helical regions are marked in blue, beta-sheets are marked in gold, position of domain/fragment boundaries are marked in red.

This study introduces *dConsensus*, a web resource for the presentation of results from automatic domain assignment algorithms. Through dConsensus, the domains assigned by seven methods are made for minimum chain length of all protein chains in the PDB (Figure 1). These assignments are available in text and graphical format alongside the assignments by the expert methods, CATH and SCOP [11] (Figure 1B). Effort was made to include all publicly available methods, these are:

• Protein Domain Parser (PDP) [12]

• DomainParser2 [13]

• PUU [14]

• DDomain [15]

• NCBI [16]

• DHcL [17]

• Dodis [18]

dConsensus also builds a consensus assignment based on the assignments by each method. Two types of approaches have been taken to generate consensus: the 'simple' approach lets each method contribute equally to the consensus (Figure 1C), while the 'weighted' approach builds the consensus by weighting the contribution of each method based on its performance, as well as considering each method's tendencies to err, as observed in a previous performance analysis [8] (Figure 1D) Additionally, the placement of domain and fragment boundaries is presented in the context of secondary structure (Figure 1E,F).

## Implementation

### Domain assignments by algorithms and expert methods

Algorithms for six of the seven methods used in this study have been implemented locally. A Java wrapper was written to sequentially run each algorithm on the entire PDB dataset and to store the results in a MySQL database. Executables for the published algorithms DDomain [15], DHcL [17], and Dodis [18] were obtained directly from the authors. Executables for both PUU [14] and PDP [12] were retrieved from the RCSB PDB [19]. The latest version of Domain Parser [13] was downloaded from http://csbl.bmb.uga.edu/downloads/#domainparser2. The NCBI-Vast method [16] had no easily installable algorithm (due to its implementation in the S language), and so the domain definitions were recovered directly from the NCBI website (e.g., for PDBid 1HTB - http://www.ncbi.nlm.nih.gov/Structure/mmdb/mmdbsrv.cgi?ShowOp=VastSum&uid=1HTB). Domain definitions for the expert methods CATH [10] and SCOP [11] were accessed from their respective text files. Version 3.2 of the CATH Domall file was downloaded from http://www.cathdb.info/wiki/doku.php?id=data:index. The design of* dConsensus *is modular; new domain assignment methods can be added by simply providing a Java wrapper for running a new method and loading the results into the database. The intention is to keep the tool up to date with new developments in the field. An Automatic update is performed once a month; ensuring assignment of domains to new proteins from the RCSB PDB.

### Generation of consensus

Methods are grouped according to the agreement among themselves (referred to as a *simple consensus*). The process begins with pair-wise comparison between two methods; the agreement between two domain assignment methods requires that the methods assign the same number of domains and fragments per domain (if a domain is fragmented). Furthermore, an 80% agreement on the placement of the boundaries is required for each domain and domain fragment, i.e., 80% of the residues should be the same for each pair of compared domains/domain fragments. If the two methods agree they form a group where one of the methods serves as a representative for the group and is used for further comparison. If methods disagree each forms its own group. Each of the remaining methods is then compared to the existing groups and is either accepted by an existing group or rejected by all, hence starting its own group. A reliability score is calculated for each group based on the percentage of contributing methods to each group. In the simplest case there is agreement among all methods and the reliability of assignment is 100%. In more complex cases several distinct competing assignments (groups) are formed. The minimum reliability score required for consensus is 40%. If none of the groups reaches that value, no consensus is reported. Should the highest score of the group be over 40%, and another group's score is less than 10% away, both groups are suggested as a potential consensus.

**Figure 2:**
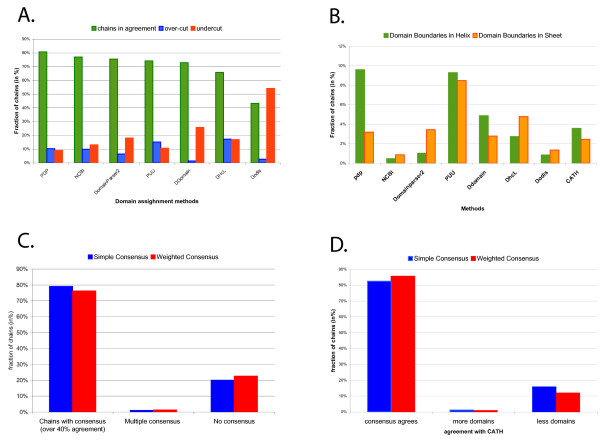
**Analysis of individual algorithmic methods and performance of consensuses**. All evaluations use 315-chain Balanced Benchmark_2 [8]. ** A**. Evaluation of domain prediction by individual methods using number of predicted domains as a sole criterion. Correct assignments are in green, over-cuts (predicting too many domains) are in red, undercuts (predicting too few domains) are in blue. **B**. Placement of domain/fragment boundaries by individual methods with respect to secondary structures. Fraction of cuts through alpha-helical structures is indicated in gold, fraction of beta-sheet cuts are indicated in green. **C**. Fraction of chains that reach consensus. Simple consensus is indicated in blue, weighted consensus indicated in red. **D**. Fraction of chains whose consensus agrees with expert consensus.

We also calculate a *weighted consensus *in which methods rather than contributing equally to the calculation of the reliability score are assigned weights based on their prior performance and specific tendencies. This is fully described in Holland et al. [8]. The initial assignments of weights for each method is based on the methods performance as determined using the number of domains as the sole criteria. Throughout this work the benchmark dataset used is the 315-chain Balanced Benchmark 2 as described in Holland et al. [8]. These weights are: PDP - 84.4%, NCBI - 81.9%, DomainParser2 - 78.1%, DDomain - 76.5%, PUU - 74.0%, DHcL - 68.3%, and Dodis - 40% (Figure 2A; for more information see Results and Discussion). The rules described in Table 1 were then applied to determine the contribution of each method.

**Table 1:** Set of rules used to determine final contributions of individual methods toward *weighted consensus*

Number of predicted domains	If the number of domains predicted by PDP and NCBI > = 4, then the weight assigned to DP is reduced by 10%
	
	Should PUU predict more domains than PDP and NCBI, downgrade PUU prediction by 10%
	
	If PDP predicts five domains or more, downgrade NCBI by 10%
Number of fragments per domain/chain	If three or more methods have at least one domain fragmented (may not be the same domain) then the weight of all methods that do not predict fragmented domains is reduced by 10%
	
	If NCBI and PDP have no fragmented domains, then the weight of all methods that predict fragmented domains is reduced by 10%

Type of Structure	If the structure is all alpha-helix (in the DSSP structure definition) and NCBI and PDP disagree on the number of domains in the chain, the weight of PDP is increased by 10%
	
	If the structure is all beta-sheet and NCBI and PDP disagree on the number of domains, the weight of PDP is increased by 10%
	
	If the structure is all beta-sheet and NCBI and PDP agree, the weight of both methods is increased by 10%
	
	If the structure is alpha-beta and NCBI and PDP agree, the weights of all methods that disagree with PDP and NCBI are reduced by 10%

### Secondary structures around domain boundaries

Domain and fragment boundaries might occur between or within secondary structure elements of the protein chain. To analyze the tendency of the algorithms to place domain/fragment boundaries inside secondary structure elements we superimposed the data from the domain/fragment boundaries with that of secondary structure elements. The secondary structure of each protein, generated using the DSSP method [14], was stored in a table within the MySQL database. To decide whether a secondary structure element is cut by the boundary we apply the following assumptions: an alpha-helix is considered to be cut by the domain/fragment boundary if the boundary falls anywhere within the helix with the exception of the two residues on either end of the helix (i.e., boundaries within the first, second, pan-ultimate or ultimate residues of the alpha-helix do not split the secondary structure). Beta-sheet structures are considered to be cut by the domain/fragment boundary if the boundary falls anywhere within the beta-sheet with the exception of the first and last residues. These considerations apply to the analysis of methods and the generation of statistics; for visualization purposes the actual secondary structure boundaries are used.

### Visualization

A platform and Web browser independent resource has been designed using Java and PHP to visualize domain assignments, consensus of the assignments (when available), as well as secondary structure elements around the domain and fragment boundaries. Domains in the protein chain are displayed using a horizontal stacked bar chart (Figure 1). Each domain is assigned its own colour. Should a domain have more than one fragment, each fragment will be the same colour to signify that it is part of the same domain.

## Results and Discussion

The process begins by entering a PDB identifier for a desired protein chain into the query form (Figure 1A). Users who wish to proceed directly to consensus should use the http://pdomains.sdsc.edu/v2/consensusform.php query form. Users who wish to view assignments by the various methods should use the http://pdomains.sdsc.edu/v2/proteinform.php query form. In the latter case the assignments by individual algorithms, as chosen by the user, are displayed in a way that allows easy comparison among methods. Assignments by the expert methods CATH and SCOP are also provided, when available, for easy comparison (Figure 1B). Domain and fragments boundaries are also displayed in a tabulated form. Consensus assignments can be reached from this page.

Consensus pages for either *simple consensus *(Figure 1C) or *weighted consensus *(Figure 1D) display the consensus assignment alongside results from CATH and SCOP. The reliability score, the reliability score interpretation and domain boundary information are also displayed. The bottom of the web page describes how the consensus was defined. The boundary analysis can be accessed from either the consensus or domain assignment page. The statistics associated with domain/fragment boundaries relative to secondary structure is presented for each method (Figure 1E). In addition users can inspect the context of individual domain/fragment boundaries (not shown), a subset of fragment boundaries (by selecting boundaries and methods of interest, not shown) or any part of the structure with all the domain boundaries within it (by selecting the range of residues in the protein chain) (Figure 1F).

The performance of seven domain assignment methods was assessed using a 315-chains expert consensus benchmark dataset referred as Balanced Benchmark 2 [8]. The overall performance of each method is measured simply as the number of correctly assigned domains (Figure 2A). The success rate of each method (fraction of correctly assigned structures) is used as the basic contribution to the weighted consensus as described in Implementation. No boundary accuracy during domain assignment is assessed in this work, however our previous work [8] indicates that 85% boundary accuracy is achieved for 95% of structures (with the exception of one method). Each method's tendency to place a domain boundary or a fragment boundary within a secondary structure, as opposed to between secondary structures, was also measured using the same 315-chain benchmark dataset. Partitioning of secondary structures by domain boundaries is specific to each method and there is no obvious correlation between overall performance of the method (Figure 2A) and tendency to partition secondary structures (Figure 2B).

The evaluation of the consensus approach indicates that consensus can be reached for almost 80% of chains in the Balanced Benchmark 2 dataset (Figure 2C). This is an encouraging result given the benchmark dataset contains mostly multi-domain proteins, typically harder to solve and hence to reach agreement. Since we require agreement among 40% of the methods, in the case of the seven methods currently involved, agreement among at least 3 methods is required in order to propel the assignment toward consensus status. Lack of consensus happens most frequently in complex structures. Thus in real situations, when no pre-selection is performed on the data, the absence of a consensus for multi-domain proteins can be expected to occur frequently. In these cases the absence of a consensus should be taken as an indication of the complexity of the structure, requiring that individual methods be looked at in detail to provide insight into how theses structures should be partitioned.

A future development will focus on an alternative consensus approach that might alleviate some frustration in difficult cases. Instead of comparing domain assignments for the entire protein chain, the reliability will be assigned to each individual boundary (domain or fragment) within the structure, by considering how many methods find the same boundary. In this case most prominent/certain partitions will be clearly assigned (having a significant reliability score), while the remaining boundaries will be less certain and left to the interpretation of the user. Providing both of the approaches to consensus will give the user a more comprehensive picture of possible partitioning.

Comparison between the simple and weighted consensus approaches favours the simple approach if we only consider the sheer number of chains which reach consensus. However, in some of these cases the consensus does not agree with CATH and SCOP predictions. There are more such cases of disagreement in the simple consensus than in the weighted consensus. The weighted consensus is slightly better than the simple consensus if we only consider cases that are in agreement with expert consensus. In either case the differences in performance between the two consensus approaches are rather small. (Figure 2C and 2D). Out of 315 chains tested there are 9 more chains in the simple consensus than in the weighted consensus (249 vs. 240 chains) and out of all consensus cases there is one more chain in the weighted consensus that agrees with expert consensus than in the simple consensus (209 vs. 208 chains). The lack of substantial difference between consensus approaches will be the focus of future improvements. It is likely that the current set of rules applied to calculate contributions to individual methods could be improved. Three of the methods involved are relatively recent (DDomain, Dodis and DHcL) and a detailed analysis along with other methods has not yet been performed.

## Conclusion

To our knowledge the dConsensus resource is the first to aggregate and provide a consensus for structure based domain methods. Not only can the results of seven algorithmic methods be viewed side by side, but the consensus among the methods can be calculated to permit the user to quickly assess possible 3D domain structure partitioning for any protein chain in the PDB. The tool is easily extendable to include new algorithmic methods should these become available.

dConsensus as included in the pDomains website should be of use to a broad audience, from students who are learning about principles of protein structure, to those who are considering improving existent methods for domain assignments, to investigators who would like a quick answer as to the possible partitioning of a new structure. The advantage of this approach is the lack of lag time typical of expert methods such as CATH and SCOP - within a month of the structure appearing in the PDB, the consensus domain assignment along with the assignments by seven algorithmic methods is available.

## Availability and requirements

• **Project name**: dConsensus

• **Project home page**: http://pdomains.sdsc.edu/dConsensus

• **Operating system(s)**: Platform independent

• **Programming language**: Java and PHP

• **Other requirements**: Java 1.3.1 or higher

• **License**: Free for non-commercial use

## Abbreviations

PDB: Protein Data Bank; PDP: Protein Domain Parser; SCOP: Structural Classification of Proteins; CATH: Hierarchical classification of protein domain structures by Class (C), Architecture (A), Topology (T) and Homologous superfamily (H); The terms 'automatic' and 'algorithmic' domain assignments are used interchangeably in this work, also by algorithm we imply algorithm used by the automatic method.

## Authors' contributions

SV and PEB conceived the work. KA wrote the code in its entirety and performed evaluation of the methods with SV's guidance. All authors wrote the manuscript and gave final approval.
